# *catena*-Poly[[(nitrato-κ^2^ *O*,*O*′)silver(I)]-μ_3_-4-pyridone-κ^3^ *O*:*O*:*O*]

**DOI:** 10.1107/S1600536809019138

**Published:** 2009-05-29

**Authors:** Xian-Ge Wu, Jun-Xia Xiao, Liang Qin

**Affiliations:** a School of Chemistry and Chemical Engineering, Zhao Qing University, Zhaoqing 526061, People’s Republic of China

## Abstract

In the title complex, [Ag(NO_3_)(C_5_H_5_NO)]_*n*_, the Ag^I^ atom is coordinated by two O atoms from two different 4-pyridone ligands and two O atoms from one nitrate anion, displaying a nearly planar coordination geometry. The O atoms of two 4-pyridone ligands bridge two symmetrically related AgNO_3_ units, forming a dimer, with an Ag⋯Ag separation of 3.680 (2) Å. Neighbouring dimers are linked into an infinite chain through weak Ag⋯O inter­actions [2.765 (2) Å], Ag⋯Ag inter­actions [3.1511 (4) Å] and π–π stacking inter­actions [centroid–centroid distance = 3.623 (4) Å]. N—H⋯O and C—H⋯O hydrogen bonds assemble these chains into a three-dimensional network.

## Related literature

For general background to hydroxy­pyridines, see: Deng *et al.* (2005 ▶); Holis & Lippard (1983 ▶); John & Urland (2006 ▶); Klausmeyer & Beckles (2007 ▶). For related structures, see: Deisenhofer & Michel (1998 ▶); Gao *et al.* (2004 ▶); Leng & Ng (2007 ▶); Li, Yan *et al.* (2005 ▶); Li, Yin *et al.* (2005 ▶); Pan & Xu (2004 ▶); Wu *et al.* (2003 ▶).
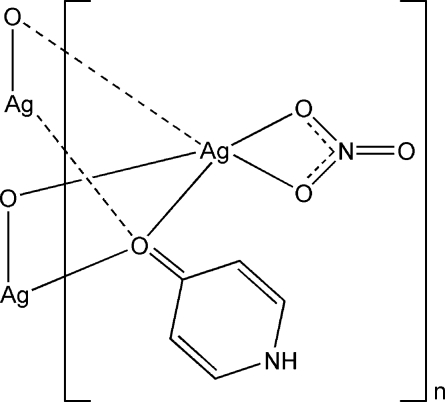


## Experimental

### 

#### Crystal data

[Ag(NO_3_)(C_5_H_5_NO)]
*M* *_r_* = 264.98
Monoclinic, 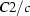

*a* = 19.3509 (7) Å
*b* = 3.6232 (1) Å
*c* = 21.2600 (8) Å
β = 102.174 (2)°
*V* = 1457.06 (9) Å^3^
*Z* = 8
Mo *K*α radiation
μ = 2.74 mm^−1^
*T* = 296 K
0.26 × 0.23 × 0.21 mm

#### Data collection

Bruker APEXII CCD diffractometer
Absorption correction: multi-scan (*SADABS*; Sheldrick, 1996 ▶) *T*_min_ = 0.508, *T*_max_ = 0.575
11458 measured reflections
1678 independent reflections
1557 reflections with *I* > 2σ(*I*)
*R*_int_ = 0.022

#### Refinement

*R*[*F* ^2^ > 2σ(*F* ^2^)] = 0.020
*wR*(*F* ^2^) = 0.054
*S* = 1.07
1678 reflections
112 parameters
1 restraint
H atoms treated by a mixture of independent and constrained refinement
Δρ_max_ = 0.50 e Å^−3^
Δρ_min_ = −0.52 e Å^−3^

### 

Data collection: *APEX2* (Bruker, 2007 ▶); cell refinement: *SAINT* (Bruker, 2007 ▶); data reduction: *SAINT*; program(s) used to solve structure: *SHELXS97* (Sheldrick, 2008 ▶); program(s) used to refine structure: *SHELXL97* (Sheldrick, 2008 ▶); molecular graphics: *SHELXTL* (Sheldrick, 2008 ▶); software used to prepare material for publication: *SHELXTL*.

## Supplementary Material

Crystal structure: contains datablocks I, global. DOI: 10.1107/S1600536809019138/hy2199sup1.cif

Structure factors: contains datablocks I. DOI: 10.1107/S1600536809019138/hy2199Isup2.hkl

Additional supplementary materials:  crystallographic information; 3D view; checkCIF report

## Figures and Tables

**Table 1:** Selected bond lengths (Å)

Ag1—O1^i^	2.3259 (15)
Ag1—O1	2.3493 (16)
Ag1—O1^ii^	2.7652 (18)
Ag1—O2	2.4132 (19)
Ag1—O3	2.5437 (18)
Ag1—Ag1^iii^	3.1511 (4)

Symmetry codes: (i) 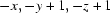; (ii) 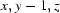; (iii) 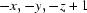.

**Table 2:** Hydrogen-bond geometry (Å, °)

*D*—H⋯*A*	*D*—H	H⋯*A*	*D*⋯*A*	*D*—H⋯*A*
C3—H3⋯O2^iv^	0.93	2.46	3.343 (3)	160
N1—H1⋯O4^v^	0.89 (3)	2.21 (2)	2.965 (3)	143 (3)
N1—H1⋯O4^iv^	0.89 (3)	2.45 (2)	3.121 (3)	133 (3)

Symmetry codes: (iv) 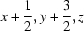; (v) 
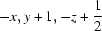
.

**Fig. 1.**
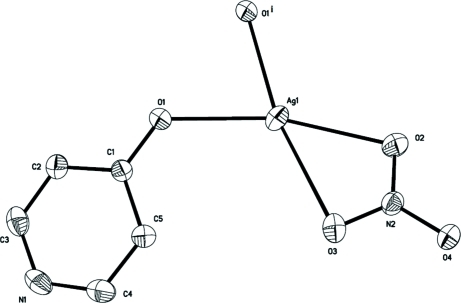
The asymmetric unit of the title compound. H atoms have been omitted for clarity. Displacement ellipsoids are drawn at the 50% probability level. [Symmetry code: (i) -x, 1-y, 1-z.]

**Fig. 2.**
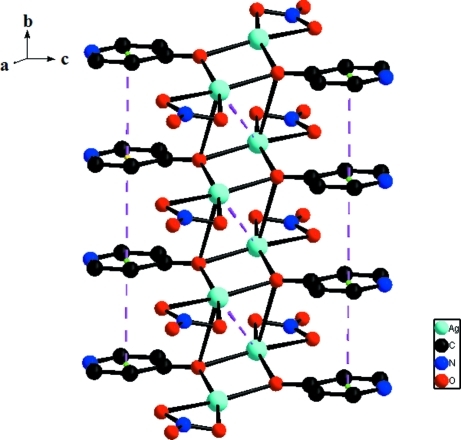
View of one-dimensional infinite chain. Dashed lines denote Ag···Ag and π–π interactions.

**Fig. 3.**
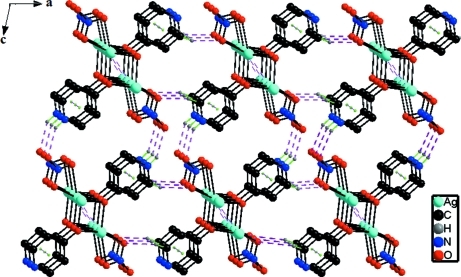
A packing view of the title compound. Hydrogen bonds are shown as dashed lines.

## supplementary crystallographic information

### Comment

Hydroxypyridines (PyOH), such as 2-, 3- and 4-PyOH, have attracted great attention in the field of crystal engineering as good candidates for the construction of supramolecular systems because they are bifunctional ligands that are not only capable of coordinating to metal ions but can also form classical hydrogen bonds as both donors and acceptors (Holis & Lippard, 1983; Klausmeyer & Beckles, 2007). 4-PyOH has two tautomers, dominated by the presence of keto form in polar solvents (Deng *et al.*, 2005; John & Urland, 2006). Thus, the protonated N atom can act as hydrogen bond donor and the PyOH uses O atom to coordinate to metal. However, the coordination chemistry of 4-PyOH ligand is still underveloped and only a few complexes have been structurally characterized in recent years (Gao *et al.*, 2004; Leng & Ng, 2007; Li, Yan *et al.*, 2005). In order to gain further insight into the metal-binding modes of the 4-PyOH ligand, we introduced Ag^I^ ion into the coordination system of the 4-PyOH ligand. In the present paper, the Ag^I^ ion only coordinates *via* the unfavoured O atom of 4-pyridone ligand, producing the title one-dimensional coordination polymer, which exhibits a three-dimensional hydrogen-bonded architecture.

The coordination environment of Ag^I^ centre is shown in Fig. 1. Each Ag^I^ atom is coordinated by two O atoms from two different 4-pyridone ligands and two O atoms from one nitrate anion (Table 1), displaying a nearly planar coordination geometry. Two 1H-pyridin-4-one ligands use their O atoms to bridge two symmetrically related AgNO_3_ units to form a dimer, with an Ag···Ag separation of 3.680 (2)Å. The adjacent dimers are linked through weak Ag···Ag interactions [3.1511 (4)Å] into a one-dimensional polymeric chain, which is also stabilized by weak Ag···O interactions [2.765 (2)Å] and intrachain π–π interactions (Fig. 2). The centroid–centroid and interplanar distances between adjacent pyridyl rings are 3.623 (4) and 3.301 (4)Å, respectively, thus indicating a weak π–π contact (Deisenhofer & Michel, 1998; Li, Yin *et al.*, 2005; Pan & Xu, 2004; Wu *et al.*, 2003). The polymeric chain shows a staircase-like array, with an Ag···Ag···Ag angle of 63.51 (4)° between three successive Ag atoms along the chain. Such an array in the chain may be explained to avoid steric hindrance. N—H···O hydrogen bonds between the ligand N atoms and the nitrate O atoms (Table 2) link adjacent chains to furnish a lamellar layer. The interlayer N—H···O and C—H···O hydrogen bonds (Table 2) further assemble the neighbouring layers, giving rise to a three-dimensional supramolecular network (Fig. 3).

### Experimental

A mixture of silver nitrate (0.17 g, 1 mmol), 4-hydroxypyridine (0.095 g, 1 mmol), NaOH (0.02 g, 0.5 mmol) and H_2_O (12 ml) was placed in a 23 ml Teflon-lined reactor, which was heated to 433 K for 3 d and then cooled to room temperature at a rate of 10 K h^-1^. The crystals obtained were washed with water and dried in air (yield 0.18 g, 69.2%).

### Refinement

C-bound H atoms were positioned geometrically and treated as riding atoms, with C—H = 0.93 Å, and with *U*_iso_(H) = 1.2*U*_eq_(C). H atom on N atom was located on difference Fourier map and refined with *U*_iso_(H) = 1.2*U*_eq_(N).

### Crystal data

**Table:**

[Ag(NO_3_)(C_5_H_5_NO)]	*F*(000) = 1024
*M**_r_* = 264.98	*D*_x_ = 2.416 Mg m^−^^3^
Monoclinic, *C*2/*c*	Mo *K*α radiation, λ = 0.71073 Å
Hall symbol: -C 2yc	Cell parameters from 3600 reflections
*a* = 19.3509 (7) Å	θ = 1.4–28°
*b* = 3.6232 (1) Å	µ = 2.74 mm^−^^1^
*c* = 21.2600 (8) Å	*T* = 296 K
β = 102.174 (2)°	Block, colorless
*V* = 1457.06 (9) Å^3^	0.26 × 0.23 × 0.21 mm
*Z* = 8	

### Data collection

**Table:**

Bruker APEXII CCD diffractometer	1678 independent reflections
Radiation source: fine-focus sealed tube	1557 reflections with *I* > 2σ(*I*)
graphite	*R*_int_ = 0.022
φ and ω scans	θ_max_ = 27.5°, θ_min_ = 2.2°
Absorption correction: multi-scan (*SADABS*; Sheldrick, 1996)	*h* = −24→24
*T*_min_ = 0.508, *T*_max_ = 0.575	*k* = −4→4
11458 measured reflections	*l* = −26→27

### Refinement

**Table:**

Refinement on *F*^2^	Primary atom site location: structure-invariant direct methods
Least-squares matrix: full	Secondary atom site location: difference Fourier map
*R*[*F*^2^ > 2σ(*F*^2^)] = 0.020	Hydrogen site location: inferred from neighbouring sites
*wR*(*F*^2^) = 0.054	H atoms treated by a mixture of independent and constrained refinement
*S* = 1.07	*w* = 1/[σ^2^(*F*_o_^2^) + (0.0276*P*)^2^ + 1.8757*P*] where *P* = (*F*_o_^2^ + 2*F*_c_^2^)/3
1678 reflections	(Δ/σ)_max_ = 0.001
112 parameters	Δρ_max_ = 0.50 e Å^−^^3^
1 restraint	Δρ_min_ = −0.52 e Å^−^^3^

### Fractional atomic coordinates and isotropic or equivalent isotropic displacement parameters (Å^2^)

**Table:**

	*x*	*y*	*z*	*U*_iso_*/*U*_eq_	
Ag1	−0.054927 (10)	0.18113 (6)	0.443081 (9)	0.04893 (9)	
C1	0.08175 (11)	0.6362 (6)	0.41324 (10)	0.0342 (4)	
C2	0.15034 (12)	0.7888 (6)	0.43328 (12)	0.0411 (5)	
H2	0.1661	0.8620	0.4758	0.049*	
C3	0.19321 (13)	0.8286 (7)	0.39055 (14)	0.0471 (6)	
H3	0.2380	0.9306	0.4040	0.057*	
C4	0.10736 (14)	0.5710 (7)	0.30792 (11)	0.0469 (5)	
H4	0.0939	0.4967	0.2652	0.056*	
C5	0.06220 (12)	0.5262 (7)	0.34795 (10)	0.0394 (4)	
H5	0.0179	0.4222	0.3325	0.047*	
H1	0.1981 (15)	0.767 (8)	0.3009 (12)	0.059*	
N1	0.17149 (12)	0.7221 (6)	0.32924 (11)	0.0486 (5)	
N2	−0.15451 (10)	−0.1759 (5)	0.34244 (9)	0.0362 (4)	
O1	0.03930 (9)	0.6001 (5)	0.45201 (7)	0.0445 (4)	
O2	−0.15517 (10)	−0.2094 (6)	0.40146 (8)	0.0543 (5)	
O3	−0.10437 (9)	−0.0108 (6)	0.32724 (9)	0.0542 (4)	
O4	−0.20310 (8)	−0.3092 (5)	0.30118 (8)	0.0480 (4)	

### Atomic displacement parameters (Å^2^)

**Table:**

	*U*^11^	*U*^22^	*U*^33^	*U*^12^	*U*^13^	*U*^23^
Ag1	0.04508 (12)	0.05614 (14)	0.04269 (12)	−0.01218 (8)	0.00275 (8)	−0.01404 (8)
C1	0.0356 (10)	0.0325 (10)	0.0360 (10)	−0.0024 (8)	0.0111 (8)	−0.0017 (8)
C2	0.0382 (11)	0.0412 (11)	0.0439 (12)	−0.0048 (9)	0.0085 (9)	−0.0019 (9)
C3	0.0353 (11)	0.0423 (13)	0.0658 (16)	−0.0008 (9)	0.0156 (11)	0.0055 (11)
C4	0.0583 (14)	0.0479 (13)	0.0378 (11)	0.0046 (11)	0.0173 (10)	0.0005 (10)
C5	0.0424 (11)	0.0417 (12)	0.0351 (10)	−0.0032 (9)	0.0101 (8)	−0.0029 (9)
N1	0.0523 (12)	0.0485 (11)	0.0532 (12)	0.0062 (9)	0.0299 (10)	0.0065 (9)
N2	0.0312 (8)	0.0395 (10)	0.0358 (9)	0.0001 (7)	0.0022 (7)	−0.0047 (7)
O1	0.0443 (8)	0.0547 (10)	0.0382 (8)	−0.0146 (7)	0.0175 (7)	−0.0098 (7)
O2	0.0548 (10)	0.0734 (12)	0.0336 (8)	−0.0131 (9)	0.0069 (8)	−0.0082 (8)
O3	0.0461 (9)	0.0610 (12)	0.0567 (10)	−0.0179 (9)	0.0132 (8)	−0.0042 (9)
O4	0.0377 (9)	0.0678 (11)	0.0360 (9)	−0.0126 (8)	0.0022 (7)	−0.0091 (8)

### Geometric parameters (Å, °)

**Table:**

Ag1—O1^i^	2.3259 (15)	C3—N1	1.339 (4)
Ag1—O1	2.3493 (16)	C3—H3	0.9300
Ag1—O1^ii^	2.7652 (18)	C4—N1	1.344 (4)
Ag1—O2	2.4132 (19)	C4—C5	1.352 (3)
Ag1—O3	2.5437 (18)	C4—H4	0.9300
Ag1—Ag1^iii^	3.1511 (4)	C5—H5	0.9300
C1—O1	1.287 (2)	N1—H1	0.89 (3)
C1—C5	1.417 (3)	N2—O3	1.239 (2)
C1—C2	1.418 (3)	N2—O4	1.240 (2)
C2—C3	1.361 (3)	N2—O2	1.263 (3)
C2—H2	0.9300	O1—Ag1^i^	2.3259 (15)
			
O1^i^—Ag1—O1	76.15 (6)	C2—C3—H3	119.7
O1^i^—Ag1—O2	118.90 (6)	N1—C4—C5	120.7 (2)
O1—Ag1—O2	163.46 (6)	N1—C4—H4	119.7
O1^i^—Ag1—O3	165.44 (6)	C5—C4—H4	119.7
O1—Ag1—O3	112.45 (5)	C4—C5—C1	120.6 (2)
O2—Ag1—O3	51.36 (5)	C4—C5—H5	119.7
O1^i^—Ag1—Ag1^iii^	58.35 (5)	C1—C5—H5	119.7
O1—Ag1—Ag1^iii^	79.67 (4)	C3—N1—C4	121.6 (2)
O2—Ag1—Ag1^iii^	113.41 (5)	C3—N1—H1	120 (2)
O3—Ag1—Ag1^iii^	133.22 (5)	C4—N1—H1	118 (2)
O1—C1—C5	121.62 (19)	O3—N2—O4	121.46 (19)
O1—C1—C2	122.0 (2)	O3—N2—O2	118.54 (19)
C5—C1—C2	116.34 (19)	O4—N2—O2	120.00 (19)
C3—C2—C1	120.3 (2)	C1—O1—Ag1^i^	127.44 (14)
C3—C2—H2	119.9	C1—O1—Ag1	127.10 (13)
C1—C2—H2	119.9	Ag1^i^—O1—Ag1	103.85 (6)
N1—C3—C2	120.6 (2)	N2—O2—Ag1	97.60 (13)
N1—C3—H3	119.7	N2—O3—Ag1	91.99 (13)

Symmetry codes: (i) −*x*, −*y*+1, −*z*+1; (ii) *x*, *y*−1, *z*; (iii) −*x*, −*y*, −*z*+1.

### Hydrogen-bond geometry (Å, °)

**Table:**

*D*—H···*A*	*D*—H	H···*A*	*D*···*A*	*D*—H···*A*
C3—H3···O2^iv^	0.93	2.46	3.343 (3)	160
N1—H1···O4^v^	0.89 (3)	2.21 (2)	2.965 (3)	143 (3)
N1—H1···O4^iv^	0.89 (3)	2.45 (2)	3.121 (3)	133 (3)

Symmetry codes: (iv) *x*+1/2, *y*+3/2, *z*; (v) −*x*, *y*+1, −*z*+1/2.
